# A novel inhibitory mechanism of MRTF-A/B on the ICAM-1 gene expression in vascular endothelial cells

**DOI:** 10.1038/srep10627

**Published:** 2015-05-29

**Authors:** Ken’ichiro Hayashi, Toshiyuki Murai, Hiroki Oikawa, Tomoyuki Masuda, Kazuhiro Kimura, Susanne Muehlich, Ron Prywes, Tsuyoshi Morita

**Affiliations:** 1 Department of RNA Biology and Neuroscience, Osaka University Graduate School of Medicine, Suita, Osaka 565-0871, Japan; 2 Department of Pathology, School of Medicine, Iwate Medical University, Yahaba-Cho, Shiwa-Gun, Iwate 028-3694, Japan; 3 Department of Ophthalmology, Yamaguchi University Graduate School of Medicine, Ube, Yamaguchi, 755-8505, Japan; 4 Walther Straub Institute of Pharmacology and Toxicology, Ludwig-Maximilians-University, Munich, Germany; 5 Department of Biological Sciences, Columbia University, New York, USA

## Abstract

The roles of myocardin-related transcription factor A (MRTF-A) and MRTF-B in vascular endothelial cells are not completely understood. Here, we found a novel regulatory mechanism for MRTF-A/B function. MRTF-A/B tend to accumulate in the nucleus in arterial endothelial cells *in vivo* and human aortic endothelial cells (HAoECs) *in vitro*. In HAoECs, nuclear localization of MRTF-A/B was not significantly affected by Y27632 or latrunculin B, primarily due to the reduced binding of MRTF-A/B to G-actin and in part, to the low level of MRTF-A phosphorylation by ERK. MRTF-A/B downregulation by serum depletion or transfection of siRNA against MRTF-A and/or MRTF-B induced ICAM-1 expression in HAoECs. It is known that nuclear import of nuclear factor−κB (NF−κB) plays a key role in ICAM-1 gene transcription. However, nuclear accumulation of NF−κB p65 was not observed in MRTF-A/B-depleted HAoECs. Our present findings suggest that MRTF-A/B inhibit ICAM-1 mRNA expression by forming a complex with NF−κB p65 in the nucleus. Conversely, downregulation of MRTF-A/B alleviates this negative regulation without further translocation of NF−κB p65 into the nucleus. These results reveal the novel roles of MRTF-A/B in the homeostasis of vascular endothelium.

Myocardin-related transcription factor A (MRTF-A) and MRTF-B are members of the myocardin family of transcriptional coactivators that activate serum response factor (SRF)^1^ and Smad^2^,^3^. MRTF-A/B are expressed in a wide variety of cells and tissues^4^,^5^ and participate in various cellular functions^3^,^6^,^7^,^8^, including epithelial–mesenchymal transition (EMT), which is closely associated with cancer progression and metastasis^9^, and tissue fibrosis^10^,^11^.

In general, MRTF-A/B are primarily located in the cytoplasm, but transiently translocate to the nucleus in response to Rho activation. In the nucleus, MRTF-A/B induce SRF- and/or Smad-mediated transcription, which enhances smooth muscle α−actin (SMα−actin) and/or snail expression, resulting in an increase in cell motility and downregulation of E-cadherin^2^,^8^.

The nuclear import and export of MRTF-A/B are regulated by importin α/β1 and CRM1 (exportin1/XPO1), respectively^12^,^13^,^14^,^15^. G-actin suppresses the interaction between MRTF-A/B and importin α/β1 and consequently inhibits the nuclear import of MRTF-A/B^12^,^13^. Furthermore, phosphorylation of MRTF-A (MAL met) by ERK at serine 454 (MAL fl at serine 546) increases its affinity for G-actin and promotes its nuclear export^16^. In some cancer cells, ERK activation is reduced because Rho pathways are constitutively activated by loss of a tumor suppressor Deleted in Liver Cancer^17^. In these cells, MRTF-A is constitutively localized in the nucleus, resulting in the activation of cell migration and proliferation. However, the mechanism of such phosphorylation-dependent nuclear export of MRTF-A is not yet fully understood.

Vascular endothelial dysfunction is critical to the initiation and development of atherosclerosis. During this process, stimulation with lipopolysaccharide (LPS), tumor necrosis factor−α (TNF−α), or oxidatively modified low-density lipoprotein (oxLDL), induces inflammatory responses by vascular endothelial cells^18^,^19^. As a result, vascular endothelial cells overexpress inflammatory cytokines, chemokines such as monocyte chemoattractant protein-1, and adhesion molecules such as intercellular adhesion molecular-1 (ICAM-1) and vascular cell adhesion molecule-1 (VCAM-1)^20^. These responses are mediated by activation of nuclear factor−κB (NF−κB), a key transcription factor for inflammatory responses^21^.

To date, several studies have established that MRTF-A plays critical roles in vascular endothelial cells, but arrived at conflicting conclusions. One study suggests that the nuclear accumulation of MRTF-A is critically important for endothelial differentiation and vascular development^22^,^23^,^24^. In contrast, other studies speculate that the external stimuli-induced accumulation of MRTF-A in the nucleus promotes either endothelial–mesenchymal transition^25^ or pathological changes in the vascular endothelium^26^. In these studies^25^,^26^, external stimuli-induced nuclear import of MRTF-A was subtle; significant amounts of MRTF-A were found in the nucleus even under non-stimulating conditions. Furthermore, the functional role of MRTF-B in vascular endothelial cells has not been determined. In this study, we investigated the subcellular localization of MRTF-A/B and its biological significance in human arterial endothelial cells.

## Results

### Subcellular localization of MRTF-A/B in arterial endothelial cells

**Figure 1:**
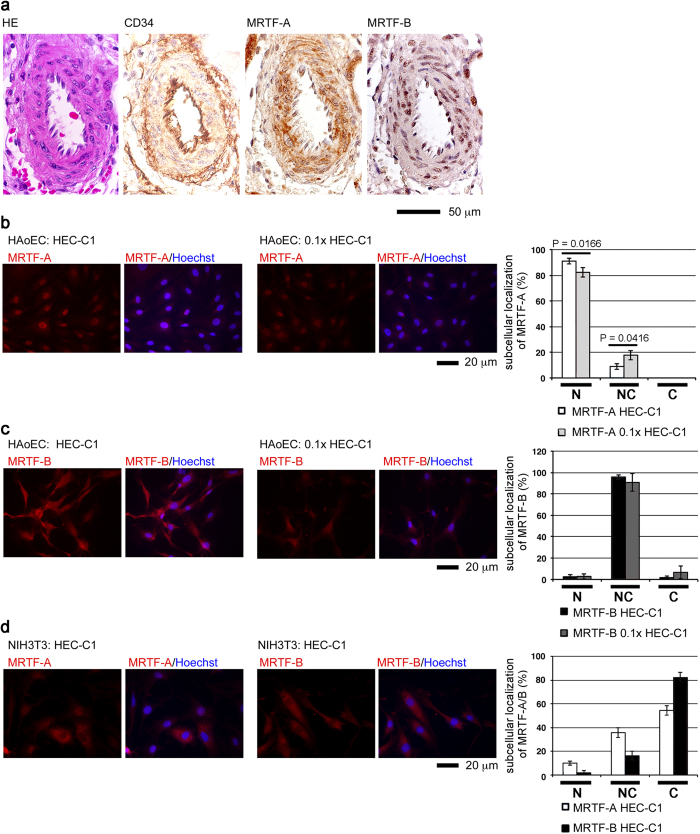
Subcellular localization of MRTF-A/B in human arterial endothelial cells. (**a**) Immunohistochemical findings of MRTF-A and MRTF-B in the endothelium of normal human renal interlobular artery. Paraffin-embedded serial transverse sections were stained with HE or an antibody against CD34, MRTF-A or MRTF-B. Representative images are shown. (**b** and **c**) HAoECs were first cultured in HEC-C1 medium. For the last 24 h, they were cultured in either HEC-C1 medium or 0.1 × HEC-C1 medium. Statistical differences were calculated using student’s t-test. (**d**) NIH3T3 cells were cultured in Dulbecco’s modified Eagle’s medium supplemented with 10% fetal calf serum and in HEC-C1 medium for the last 24 h. Cells were stained with anti-MRTF-A antibody or anti-MRTF-B antibody (red) and Hoechst 33258 (blue). Representative images from at least three independent experiments are shown. Images were quantified as described in Materials and Methods: nuclear-specific localization (N), diffuse distribution in the nucleus and the cytoplasm (NC), and cytoplasmic localization (C) (left column).

We first analyzed the subcellular localization of MRTF-A and MRTF-B in normal human arterial endothelium (Fig. 1a). For immunohistochemical examination of the expression of MRTF-A and MRTF-B, we morphologically selected normal renal arteries (interlobular arteries) by staining with hematoxylin-eosin (HE). Immunostaining for MRTF-A and MRTF-B showed positive reactions in the medial layer. Interestingly, the arterial endothelium expressing CD34 were also positive for MRTF-A and MRTF-B. The expression of MRTF-A and MRTF-B was mainly observed in the nucleus.

We then examined the subcellular localization of MRTF-A/B in human aortic endothelial cells (HAoECs) for detailed investigation of the biological roles of MRTF-A/B. It is well known that serum-induced Rho activation promotes the nuclear import of MRTF-A/B in cultured cells. In our previous study using COS-7 cells^14^ and NIH3T3 cells^27^, we clearly demonstrated that the majority of MRTF-A is localized in the cytoplasm under low-serum culture conditions, and that the nuclear accumulation of MRTF-A occurs transiently just after serum stimulation. Thereafter, nuclear MRTF-A is gradually exported to the cytoplasm. Thus, MRTF-A is mainly localized in the cytoplasm 24 h after serum stimulation. To our surprise, the majority of MRTF-A was found in the nucleus in HAoECs cultured in HEC-C1 medium containing 10% fetal calf serum, acidic fibroblast growth factor, and heparin (see Materials and Methods) (92.9 ± 4.1%). Nuclear accumulation of MRTF-A was also observed in HAoECs cultured in 0.1× HEC-C1 medium, diluted HEC-C1 medium by basal medium without fetal calf serum and other additional factors [see Materials and Methods]) (81.9 ± 4.0%) (Fig. 1b). However, the nuclear accumulation of MRTF-A was only slightly decreased under such culture conditions. MRTF-B was diffusely distributed in the nucleus and throughout the cytoplasm (95.7 ± 2.0% in HEC-C1 medium and 90.7 ± 8.2% in 0.1× HEC-C1 medium) (Fig. 1c). Significant difference in the distribution of MRTF-B was not observed between both culture conditions. The staining intensities of both proteins were, however, weak in HAoECs cultured in 0.1× HEC-C1 medium (Fig. 1b,c). Similar expression patterns of MRTF-A/B were also observed when HAoECs were cultured in further diluted medium (0.03× HEC-C1 medium). These results suggest that serum and the additional factors in HEC-C1 medium do not affect the nuclear localization of MRTF-A/B in HAoECs. To exclude the possibility that the nuclear localization of MRTF-A/B was brought about by the additional factors aside from serum in HEC-C1 medium, we further investigated the subcellular localization of MRTF-A/B in NIH3T3 cells cultured in HEC-C1 medium (Fig. 1d). In the majority of the cells, MRTF-A/B were primarily found in the cytoplasm (54.4 ± 4.2% for MRTF-A and 82.1 ± 4.8% for MRTF-B). These results coincided well with the subcellular localization of MRTF-A in NIH3T3 cells cultured in Dulbecco’s modified Eagle’s medium supplemented with 10% fetal calf serum^27^, suggesting that the additional factors aside from serum in HEC-C1 medium do not affect the nuclear localization of MRTF-A. To further confirm the above possibility, we analyzed the subcellular localization of MRTF-A/B in HAoECs cultured in Dulbecco’s modified Eagle’s medium supplemented with 10% or 0.3% fetal calf serum without acidic fibroblast growth factor and heparin. We chose this low-serum medium to suppress Rho activation just after serum stimulation and to minimize the inhibitory effect on cell viability caused by serum starvation. Similar subcellular localization of MRTF-A/B were observed in HAoECs cultured in Dulbecco’s modified Eagle’s medium supplemented with 10% or 0.3% fetal calf serum (Supplementary Fig. 1a,b), suggesting that the nuclear accumulation of MRTF-A/B is not caused by other additional factors aside from serum in HEC-C1 medium. These results indicate that in arterial endothelial cells, MRTF-A/B tend to accumulate in the nucleus *in vivo* and *in vitro* and that their expression levels decrease under low-serum culture conditions. However, the expression of EMT marker, SMα−actin was not detected.

**Figure 2:**
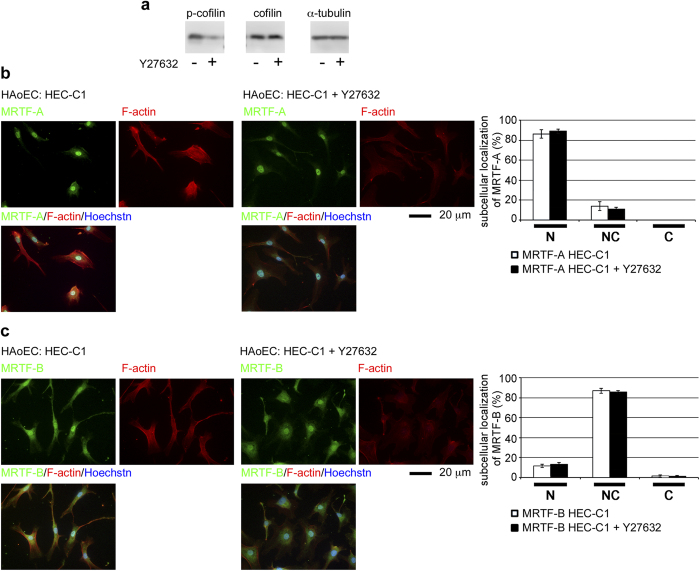
Effects of Y27632 on the subcellular localization of MRTF-A/B in HAoECs. HAoECs were cultured in HEC-C1 medium. For the last 12 h, they were treated with vehicle or 10 μM Y27632. (**a**) Whole cell lysates were subjected to IB analyses with the indicated antibodies. α↕tubulin was used as a loading control. (**b** and **c**) Cells were stained either with anti-MRTF-A antibody or anti-MRTF-B antibody (green), phalloidine-Alexa 568 (red), and Hoechst 33258 (blue). Representative images from at least three independent experiments are shown. Images were quantified as described in the legend for Fig. 1.

To further address the role of actin dynamics in the nuclear accumulation of MRTF-A/B in HAoECs, we analyzed their subcellular localization in the cells treated with either the Rho-kinase inhibitor Y27632 or the actin polymerization inhibitor latrunculin B (LatB). To our surprise, the nuclear accumulation of MRTF-A/B was not inhibited by Y27632; treatment with Y27632 decreased the phosphorylation of cofilin at serine 3 and F-actin but did not suppress the nuclear localization of MRTF-A/B (Fig. 2). Similar results were found in HAoECs cultured in LatB-containing medium. Treatment with LatB significantly decreased F-actin staining, but only slightly affected the nuclear localization of MRTF-A/B (Supplementary Fig. 1c,d). These results suggest that the nuclear accumulation of MRTF-A/B is resistant to an increase in G-actin.

### Molecular mechanism for the nuclear accumulation of MRTF-A/B in HAoECs

**Figure 3:**
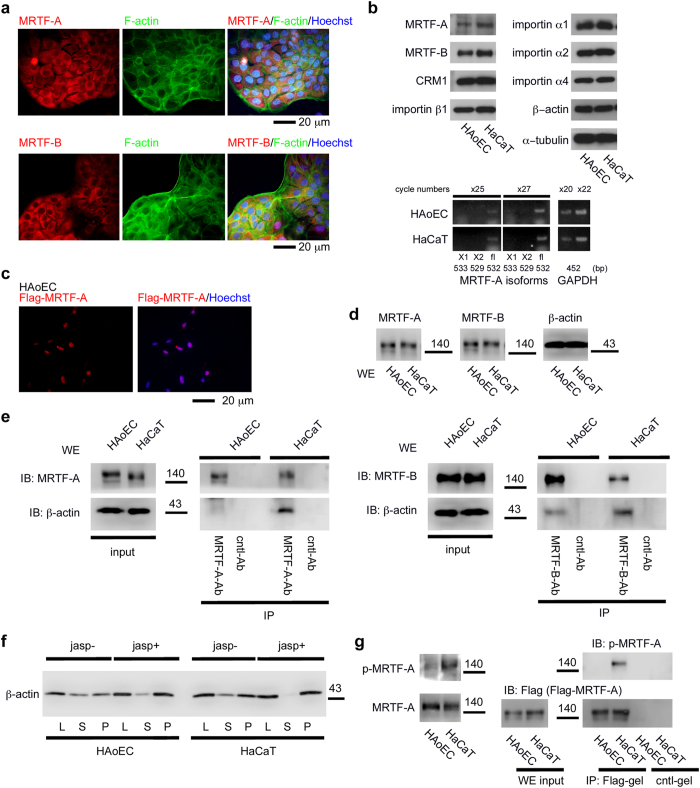
Properties of MRTF-A/B in HAoECs and HaCaT cells. HaCaT cells were cultured in Dulbecco’s modified Eagle’s medium supplemented with 10% fetal calf serum and in HEC-C1 medium for the last 24 h. HAoECs were cultured in HEC-C1 medium. (**a**) HaCaT cells were stained with anti-MRTF-A antibody or anti-MRTF-B antibody (red), phalloidine-Alexa 488 (green), and Hoechst 33258 (blue). Representative images from at least three independent experiments are shown. (**b**) IB analysis shows the expression levels of MRTF-A/B and proteins involved in their nuclear import and export in HAoECs and HaCaT cells (upper panel). Whole cell lysates (WL) were subjected to IB with the indicated antibodies. α□tubulin was used as a loading control. RT-PCR analyses for monitoring the expression of MRTF-A isoforms (MAL fl [fl], variant X1 [X1], and variant X2 [X2]) in HAoECs and HaCaT cells (lower panel). PCR products were sampled at the indicated time points after 20 to 27 cycles and separated on 1.2% agarose gels. (**c**) Nuclear accumulation of exogenously expressed MRTF-A in HAoECs. HAoECs expressing Flag-tagged mouse MRTF-A (MAL fl) were stained with anti-Flag antibody (red) and Hoechst 33258 (blue). (**d** and **e**) IB analysis shows the expression levels of MRTF-A/B and β−actin in the whole cell extracts (WE) from HAoECs and HaCaT cells (**d**). The respective WE were subjected to IP analyses with a control antibody (cntl-Ab) or either anti-MRTF-A antibody (**e** left panel) or anti-MRTF-B antibody (**e** right panel). The IP/IB analyses were performed with the indicated antibodies. Positions of molecular weight markers (kDa) are indicated between the IB panels. (**f**) Actin fractionation. HAoECs and HaCaT cells were either left untreated (jasp-) or treated with jasplakinolide (0.3 μM; jasp+) for the last 60 min. The respective lysates (L) were separated into supernatant (S) and pellet (P) fractions by centrifugation, and they were subjected to IB with anti-β−actin antibody. (**g**) ERK phosphorylation of MRTF-A. WL from the indicated cells were subjected to IB with anti-MRTF-A or the phospho-specific MRTF-A antibody (left panel). WE from respective cells expressing Flag-tagged mouse MRTF-A (MAL fl) were subjected to IP/IB analysis with the indicated antibodies (right panel).

To further investigate the mechanism for the nuclear accumulation of MRTF-A/B in HAoECs, we searched for normal human cells in which MRTF-A/B are localized in the cytoplasm. In human keratinocyte HaCaT cells, MRTF-A/B were predominantly found in the cytoplasm and F-actin staining was particularly strong in the plasmalemmal undercoat (Fig. 3a). There were no significant differences in the expression levels of proteins involved in the nuclear import and export of MRTF-A/B between HAoECs and HaCaT cells (Fig. 3b, upper panel). RT-PCR analyses revealed that in both cells, only the transcript for full-length MRTF-A (MAL fl) [GenBank: AB037859.2] was expressed; transcripts for other MRTF-A isoforms (MAL BSAC/MKL1 transcript variant X1) [NCBI Reference Sequence: XM_005261691.1] and MKL1 transcript variant X2 [NCBI Reference Sequence: XM_005261692.1] were not expressed (Fig. 3b, lower panel). These results suggest that MAL fl is the major MRTF-A subtype in both cells. We confirmed that exogenously expressed mouse MRTF-A (MAL fl) in HAoECs was also localized in the nucleus (Fig. 3c).

We then examined the binding levels of MRTF-A/B to β−actin by immunoprecipitation (IP) analyses using the respective whole cell extracts from HAoECs and HaCaT cells (Fig. 3d,e). Interesting, β−actin binding to MRTF-A/B in HAoECs was markedly reduced compared to that in HaCaT cells, indicating that MRTF-A/B are unlikely to be associated with G-actin in HAoECs. Analysis of F- and G-actin ratios revealed that the G-actin pools in the two cell types were comparable (Fig. 3f).

**Figure 4:**
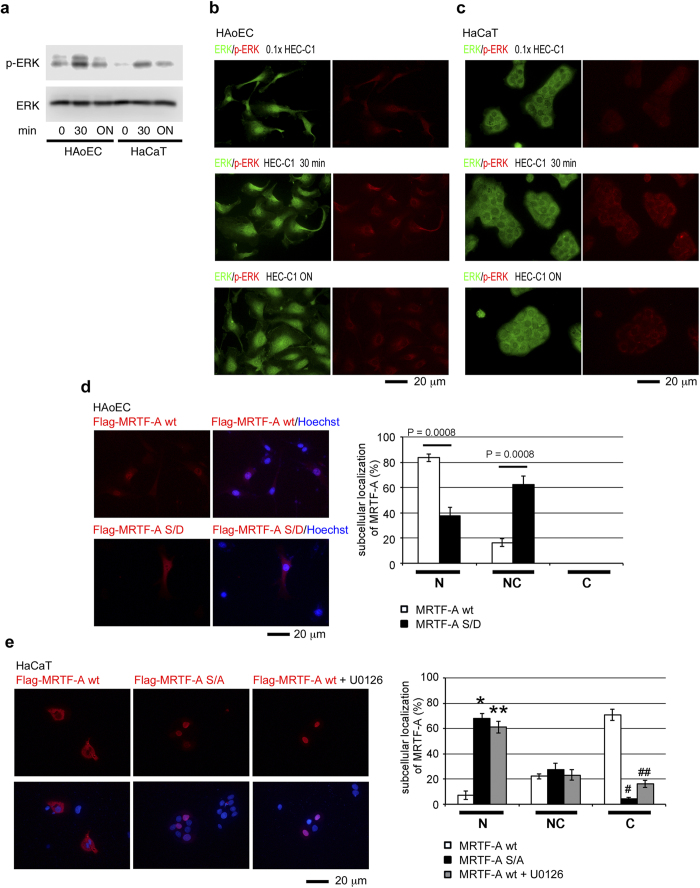
Relation between ERK activation and subcellular localization of MRTF-A in HAoECs and HaCaT cells. (**a**,**b**, and **c**) HAoECs and HACaT cells were cultured in 0.1× HEC-C1 medium for 24 h, and then they were restimulated with HEC-C1 medium for 30 min and overnight (ON). Whole cell lysates at the indicated time points were subjected to IB with the indicated antibodies (**a**). HAoECs (**b**) and HaCaT cells (**c**) were stained with anti-ERK antibody (green) and anti-p-ERK antibody (red). Representative images from at least three independent experiments are shown. (**d** and **e**) Subcellular localization of exogenously expressed wild-type (wt) and mutant MRTF-As in HAoECs and HaCaT cells. HAoECs (**d**) and HaCaT cells (**e**) were transfected with each of the indicated Flag-tagged MRTF-A expression plasmids and were cultured in HEC-C1 medium containing either vehicle or 10 μM U0126 for 24 h. Cells were stained with anti-Flag antibody (red) and Hoechst 33258 (blue). Representative images from at least three independent experiments are shown. Images were quantified as described in the legend for Fig. 1. Statistical differences were calculated using student’s t-test. *P = 8.2580 × 10^−5^, **P = 0.0002, ^#^P = 8.4164 × 10^−7^, and ^##^P = 1.5908 × 10^−5^ *versus* the values of MRTF-A wt in the respective localization categories (**e**).

Phosphorylation of MRTF-A plays a critical role in the subcellular localization of MRTF-A and SRF-dependent transcription^16^,^17^,^28^. Notably, MRTF-A phosphorylation by ERK increases its affinity for G-actin and promotes the nuclear export of MRTF-A^16^. To examine the phosphorylation state of MRTF-A by ERK in HAoECs and HaCaT cells, we performed immunoblot (IB) analysis with the phospho-specific MRTF-A ([p-MKL1] serine 454 MAL met/serine 546 MAL fl) antibody^16^. We found a significant difference in the phosphorylation state of MRTF-A; the ERK phosphorylation site was markedly phosphorylated in HaCaT cells but not in HAoECs (Fig. 3g). These findings raise the question of how the reduced phosphorylation of MRTF-A by ERK occurs in HAoECs. To address this issue, we investigated the activation of ERK by monitoring the levels of phosphorylated ERK (p-ERK) in HAoECs and HaCaT cells. ERK activation was similarly observed in both cells (Fig. 4a). We further investigated the cellular distribution of ERK and p-ERK. In HAoECs, ERK was diffusely distributed in the nucleus and throughout the cytoplasm (Fig. 4b). On the other hand, ERK was mainly distributed in the cytoplasm of HaCaT cells (Fig. 4c). However, p-ERK was predominantly found in the cytoplasm in both cells (Fig. 4b,c).

We also validated the concept that ERK phosphorylation of MRTF-A promotes its nuclear export^16^. To address this issue, we prepared mutant mouse MRTF-A proteins in which the key ERK phosphorylation site (serine 549 mouse MAL fl) was changed to alanine (S/A, non-phosphorylation mutant) or aspartate (S/D, pseudo-phosphorylation mutant). We then examined the interaction between CRM1 and the wild-type and these mutant MRTF-As *in vitro* (Supplementary Fig. 2). In the absence of constitutively GTP-bound Ran (RanQ69L), CRM1 binding to MRTF-A was hardly detected^14^,^15^. Compared with wild-type MRTF-A, un-phosphorylated mutant (MRTF-A S/A) exhibited reduced binding to CRM1. In contrast, the CRM1-binding capacity of pseudo-phosphorylated mutant (MRTF-A S/D) significantly increased, suggesting that ERK phosphorylation of MRTF-A enhances its binding to CRM1 (Supplementary Fig. 2a). Previous results indicated that the binding of G-actin to MRTF-A promotes its nuclear export^29^ and MRTF-A phosphorylation by ERK increases its binding affinity for G-actin^16^. Contrary to these findings, our recent *in vitro* protein-protein interaction analysis demonstrated that CRM1 binding to MRTF-A is inhibited by G-actin^14^. We speculated that phosphorylation of MRTF-A by ERK confers resistance to such inhibition. To address this issue, we examined the MRTF-A S/D binding to CRM1 in the absence or presence of G-actin. Unlike wild-type MRTF-A (Supplementary Figure 2b), MRTF-A S/D did not show reduced binding to CRM1 in the presence of G-actin (Supplementary Figure 2c). Thus, enhanced nuclear export of ERK-phosphorylated MRTF-A^16^ would be partially due to its increased binding to CRM1. To confirm this prediction *in vivo*, we examined the interaction between CRM1 and either MRTF-A or MRTF-B in HAoECs and HaCaT cells. However, such interactions could not be detected by IP/IB analyses. This is probably due to the transient binding of CRM1 to MRTF-A/B and the low levels of expression of MRTF-A/B in these cells. Thus, we further analyzed the subcellular localization of exogenously expressed mutant MRTF-As in both cells. In HAoECs, wild-type MRTF-A was predominantly found in the nucleus (83.6 ± 3.1%), whereas pseudo-phosphorylated mutant (MRTF-A S/D) was found either exclusively in nucleus (37.5 ± 6.5%) or both in the nucleus and the cytoplasm (62.5 ± 6.5%) (Fig. 4d). In HaCaT cells, wild-type MRTF-A was primarily localized in the cytoplasm (67.2 ± 1.1%), while un-phosphorylated mutant (MRTF-A S/A) was mainly found in the nucleus (67.9 ± 3.9%). Treatment with the ERK inhibitor U0126 markedly induced nuclear accumulation of MRTF-A (Fig. 4e and Supplementary Fig. 3). These results support the reliability of nuclear localization of exogenously expressed MRTF-A S/A.

### Inhibition of ICAM-1 expression by MRTF-A/B

**Figure 5:**
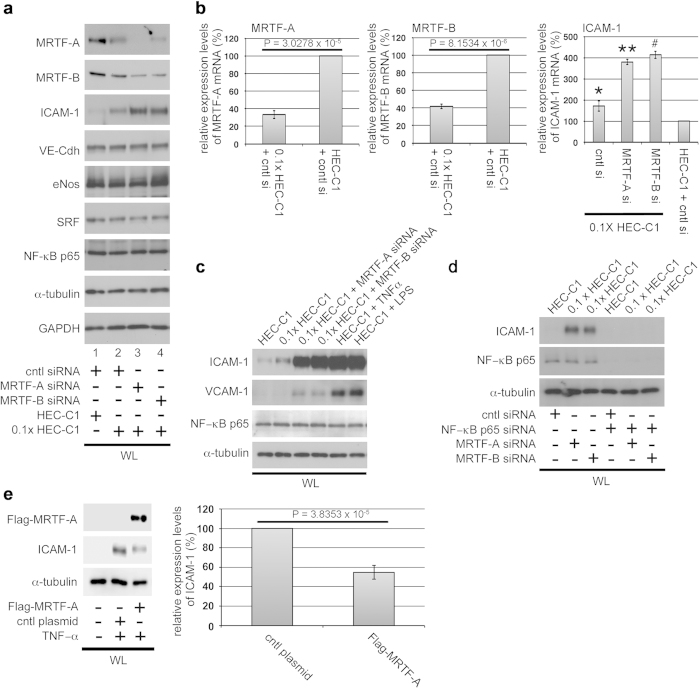
Upregulation of ICAM-1 expression in MRTF-A/B-depleted HAoECs. (**a**) Expression levels of MRTF-A/B, ICAM-1, endothelial cell markers, and transcription factors. HAoECs were transfected with control (cntl) siRNA or either anti-MRTF-A siRNA or anti-MRTF-B siRNA and were cultured for 2 days in HEC-C1 medium. For the last 24 h, they were cultured in HEC-C1 medium (lane 1) or in 0.1× HEC-C1 medium (lanes 2–4). Whole cell lysates (WL) were subjected to IB with the indicated antibodies. α−tubulin and GAPDH were used as loading controls. (**b**) Quantitative real-time RT-PCR analyses for MRTF-A/B and ICAM-1 mRNA expressions in HAoECs. Expression levels of the indicated mRNAs were normalized to GAPDH mRNA. The levels of the respective mRNAs in cntl siRNA-transfected HAoECs cultured in HEC-C1 medium were set at 100% (means ± SEMs of three independent experiments). (**c**) HAoECs were transfected with cntl siRNA (no label) or either anti-MRTF-A siRNA or anti-MRTF-B siRNA and were cultured for 2 days in HEC-C1 medium. For the last 24 h, these cells were cultured in each of the following media: HEC-C1, 0.1× HEC-C1, and HEC-C1 containing TNF−α (1 ng/ml) or LPS (0.1 μg/ml). (d) HAoECs were transfected with the indicated siRNAs and were cultured as described above. For the last 24 h, these cells were cultured under the indicated conditions. WL were subjected to IB with the indicated antibodies. α−tubulin was used as a loading control (c and d). (**e**) HAoECs were transfected with cntl plasmid or Flag-MRTF-A expression plasmid and were cultured for 2 days in HEC-C1 medium. For the last 20 h, they were treated with vehicle or TNF−α (1 ng/ml). WL were subjected to IB with the indicated antibodies. Representative results from three independent experiments are shown. The level of ICAM-1 protein in cntl plasmid-transfected HAoECs cultured in TNF−α-containing HEC-C1 medium was set at 100% (means ± SEMs of three independent experiments) (**e**). Statistical differences were calculated using student’s t-test. *P = 0.0172, **P = 5.4762 × 10^−6^, and ^#^P = 1.1850 × 10^−5^ *versus* the value from cntl siRNA-transfected cells in HEC-C1 medium (**b** right panel).

To confirm the downregulation of MRTF-A/B in HAoECs cultured in 0.1× HEC-C1 medium (Fig. 1b,c), we analyzed the expression levels of MRTF-A/B, markers of endothelial cells (VE-cadherin [VE-Cdh] and endothelial nitric oxide synthase [eNos]), and transcription factors by immunoblotting (Fig. 5a). MRTF-A/B expression decreased, while ICAM-1 expression (a biomarker of endothelial dysfunction) increased in HAoECs cultured in 0.1× HEC-C1 medium. ICAM-1 is a ligand for lymphocyte function-associated antigen-1, a cell surface receptor found on leukocytes that plays a critical role in the transmigration of leukocytes into the vessel wall^30^. The expression levels of VE-Cdh, eNos, SRF, and NF−κB p65 did not change under both culture conditions with complete growth and low-serum media (Fig. 5a, lanes 1 and 2). ICAM-1 expression was further enhanced by siRNA-mediated knockdown of either MRTF-A or MRTF-B, whereas expression of the other biomarkers was not (Fig. 5a, lanes 3 and 4). Of interest, unlike other cells, knockdown of either MRTF-A or MRTF-B resulted in the downregulation of both MRTF-A and -B in HAoECs (Fig. 5a, lanes 2–4 and Supplementary Fig. 4), suggesting that the expression levels of MRTF-A and MRTF-B are mutually dependent. Compared to MRTF-A and MRTF-B expression levels in HEC-C1 medium, RT-PCR analyses indicated that their expression levels in HAoECs cultured in 0.1× HEC-C1 medium decreased to 33.5 ± 4.5% and 41.8 ± 2.8%, respectively (Fig. 5b, left and middle columns). ICAM-1 mRNA expression increased to 171.3 ± 25.7% after serum depletion and further increased to 378.9 ± 12.2% and 413.3 ± 16.6% after the knockdown of MRTF-A and MRTF-B, respectively (Fig. 5b, right column). These results indicate that ICAM-1 upregulation is closely associated with MRTF-A/B downregulation. TNF−α- or LPS-induced activation of ICAM-1 gene transcription critically depends on NF−κB^31^. Thus, we compared ICAM-1 expression induced by MRTF-A/B downregulation with that after TNF−α or LPS stimulation. Considerable ICAM-1 expression was found in both siRNA-mediated MRTF-A/B knockdown or stimulation with TNF−α or LPS. VCAM-1 expression (a biomarker of endothelial dysfunction) was also induced by MRTF-A/B downregulation, although these effects were modest compared to those observed after TNF−α or LPS stimulation. However, NF−κB p65 expression levels were similar among all of the culture conditions we examined (Fig. 5c). siRNA-mediated knockdown of NF−κB p65 prevented the induction of ICAM-1 expression in MRTF-A/B-depleted HAoECs (Fig. 5d). These results suggested the functional involvement of NF−κB p65 in these cellular events. Furthermore, forced expression of MRTF-A reduced TNF−α-induced ICAM-1 expression (Fig. 5e), suggesting an inhibitory role of MRTF-A in the expression of ICAM-1 in HAoECs.

### Differences between MRTF-A/B depletion-induced ICAM1 expression and TNF−α- or LPS-induced ICAM1 expression

**Figure 6:**
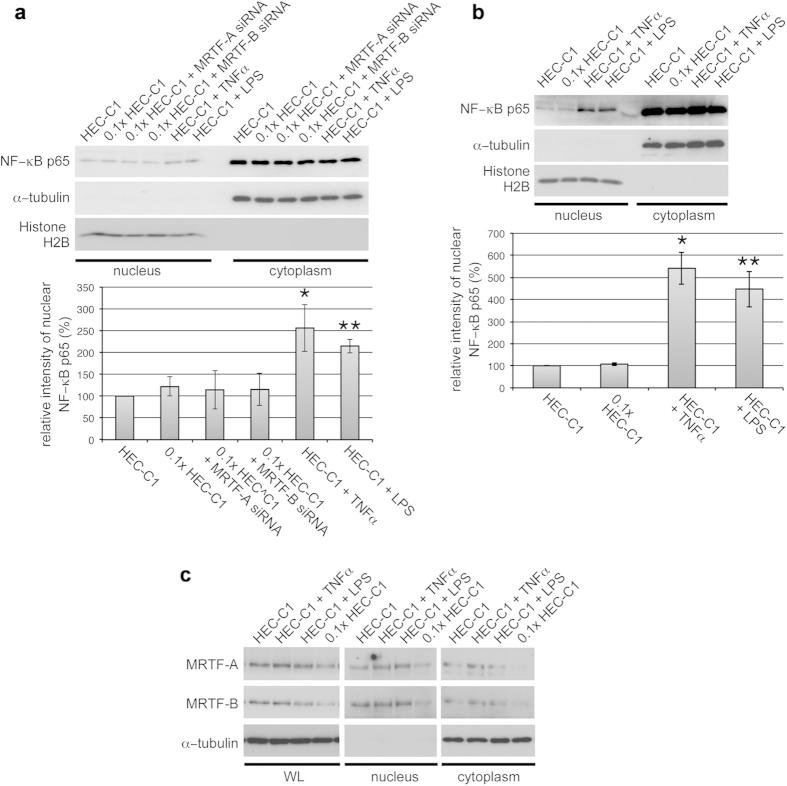
Subcellular localization of NF−κB p65 in MRTF-A/B-depleted and TNF −α - or LPS-stimulated HAoECs. HAoECs were transfected with control siRNA (no label) or either anti-MRTF-A siRNA or anti-MRTF-B siRNA and were cultured in HEC-C1 medium. For the last 24 h (a and c) or 1 h (**b**) these cells were cultured under the indicated conditions, and their whole cell lysates (WL) and/or nuclear and cytoplasmic fractions were subjected to IB with the indicated antibodies as described in Materials and Methods. Histone H2B and α−tubulin were used as loading controls for the nuclear and cytoplasmic fractions, respectively. Representative results from three independent experiments are shown. IB signal intensities for nuclear NF−κB p65 were quantified. Percentages indicate the relative intensities of nuclear NF−κB p65 normalized by the intensity in HAoECs cultured in HEC-C1 medium without any treatment, which were set at 100% (means ± SEMs of the results from three independent experiments). Statistical differences were calculated using student’s t-test. *P = 0.0004 and **P = 4.8090 × 10^−7^ *versus* the value from control siRNA-transfected cells in HEC-C1 medium. (**a**) *P = 1.2593 × 10^−7^ and **P = 1.4400 × 10^−6^ *versus* the value from control siRNA-transfected cells in HEC-C1 medium (**b**).

We investigated the distribution of NF−κB p65 in the nuclear and cytoplasmic fractions. TNF−α- or LPS-stimulation triggered an approximately 2-fold increase in NF−κB p65 nuclear localization in HAoECs cultured under the different conditions for 24 h (Fig. 6a). Similarly, short-term (1 h) stimulation with TNF−α or LPS induced an approximately 5-fold increase in NF−κB p65 nuclear localization (Fig. 6b). However, NF−κB p65 nuclear localization in HAoECs cultured under MRTF-A/B-depleted and/or low-serum conditions was equivalent to that in culture with complete growth medium (Fig. 6a,b). In TNF−α- or LPS-stimulated HAoECs, MRTF-A/B expression levels did not decrease, and their subcellular localizations were not affected (Fig. 6c). These results demonstrate that MRTF-A/B depletion and/or serum depletion do not induce nuclear accumulation of NF−κB p65. In contrast, stimulation with TNF−α or LPS causes nuclear accumulation of NF−κB p65 but does not affect the expression levels and subcellular localization of MRTF-A/B. Thus, additional nuclear import of NF–κB p65 appears to be unnecessary for the upregulation of ICAM-1 gene expression under MRTF-A/B-depleted and/or low-serum culture conditions.

### Effects of MRTF-A/B depletion on the interactions between HAoECs and leukocytes

We first analyzed the cell surface expression of ICAM-1 in MRTF-A/B-depleted HAoECs by flow cytometry. Compared with HAoECs transfected with control siRNA, ICAM-1 cell surface expression increased in HAoECs transfected with either anti-MRTF-A siRNA or anti-MRTF-B siRNA (Supplementary Fig. 5a,b). We then performed cell adhesion assays. siRNA-mediated knockdown of either MRTF-A or MRTF-B significantly increased the interaction between HAoECs and leuokocytes (Supplementary Figure 5c). These results indicate that MRTF-A/B depletion is closely related to endothelial cell injury.

### Suppression of NF−κB-mediated ICAM-1 gene transcription by MRTF-A/B

It is well documented that ICAM-1 gene transcription is positively regulated by NF−κB. NF−κB p65 homodimer binds to the NF−κB binding site located in the human ICAM-1 gene promoter region (-191 to -176)^32^. To investigate the transcriptional regulation of the ICAM-1 gene by MRTF-A/B, we performed a chromatin immunoprecipitation (ChIP) assay using an antibody against NF−κB p65. ChIP-PCR showed that siRNA-mediated knockdown of MRTF-A/B significantly increased the binding of NF−κB p65 to the NF−κB-binding site in the ICAM-1 gene promoter (Supplementary Figure 6a). This suggests that MRTF-A/B is involved in suppressing NF−κB-mediated ICAM-1 gene transcription. Tang *et al.* and Wang *et al.* reported that myocardin and MRTF-A directly bind to NF−κB p65 and inhibited NF−κB-dependent gene expression^33^,^34^. Based on these findings, we speculated that MRTF-A/B would directly inhibit the NF−κB-mediated ICAM-1 gene expression by forming complexes with NF−κB p65 in HAoECs. To confirm this hypothesis, we first confirmed that NF−κB p65 forms a complex with MRTF-A or MRTF-B (Supplementary Figure 6b). We then examined the effects of MRTF-A/B on the interaction between NF−κB p65 and the NF−κB-binding site in the ICAM-1 gene promoter region using a Dynabeads-coupled NF−κB probe (Dynabeads-NF−κB oligos). In this analysis with *in vitro* translated proteins, NF−κB p65-probe interaction was markedly inhibited in the presence of MRTF-A or MRTF-B. This is due to inactivation of NF−κB p65 by forming a complex with either MRTF-A or MRTF-B (Supplementary Figure 6c). Similar results were also observed in the analyses using whole cell extracts from HAoECs transfected with either anti-MRTF-A siRNA or anti-MRTF-B siRNA (Supplementary Figure 6d). The interaction between endogenous NF−κB p65 and the probe was limited to cells in which the expression levels of MRTF-A and MRTF-B were decreased. Moreover, such interaction was markedly suppressed by free NF−κB oligos. These results support the possibility that MRTF-A/B inhibit NF−κB p65 binding to the ICAM-1 gene promoter by forming complexes with NF−κB p65 in HAoECs.

## Discussion

**Figure 7:**
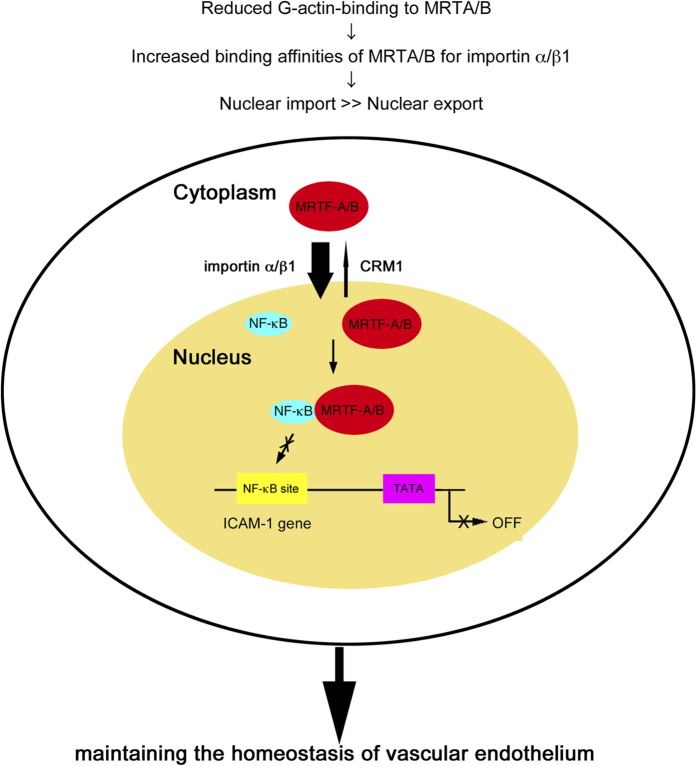
Molecular mechanism for the constitutive nuclear accumulation of MRTF-A/B and their protective roles against endothelial cell injury. Our present findings are schematically summarized.

Our present findings are schematically summarized in Fig. 7; MRTF-A/B tend to accumulate in the nucleus because of their reduced binding to G-actin and play a critical role in maintaining the homeostasis of vascular endothelium.

The extracellular stimuli-induced nuclear accumulation of MRTF-A in HAoECs^26^ and mouse pancreatic microvascular endothelial cells^25^ was recently reported. In those studies, MRTF-A expression levels significantly increased after stimulation with oxLDL or transforming growth factor−β, and nuclear accumulation of MRTF-A is concomitantly enhanced with an increase in MRTF-A expression. In our present study, MRTF-A/B were mainly observed in the nuclei of human arterial endothelial cells *in vivo* and in cultured HAoECs (Fig. 1). Nuclear localization of MRTF-A was not affected even under low-serum culture conditions, although the expression level was reduced. Similarly, the MRTF-B expression pattern, a diffuse distribution in the nucleus and throughout the cytoplasm, remained unchanged, although its expression level was also decreased by serum depletion. The nuclear localization of MRTF-A/B was resistant to an increase in G-actin (Fig. 2 and Supplementary Fig. 1c,d). These properties are primarily due to reduced binding of MRTF-A/B to G-actin (Fig. 3d,e) because G-actin-bound MRTF-A/B exhibit reduced binding to importin α/β1^12^,^13^. However, the G-actin pool in HAoECs was equivalent to that in HaCaT cells (Fig. 3f). At present, we cannot fully elucidate how MRTF-A/B exhibit low binding to G-actin in HAoECs. One possible explanation is that the G-actin-binding sites of MRTF-A/B are masked by a protein factor bound to MRTF-A/B or a posttranslational modification. Because it is known that MRTF-A interacts with several protein factors^35^ or undergoes sumoylation^36^. Reduced phosphorylation of MRTF-A by ERK (Fig. 3g) could be another possibility because ERK phosphorylated MRTF-A shows an increased affinity for G-actin^16^. The difference in ERK phosphorylation of MRTF-A in HAoECs and HaCaT cells could be due to limited activation of ERK in the cytoplasm in both cells (Fig. 4b,c). In HAoECs, MRTF-A was unlikely to be phosphorylated by ERK because the localization of MRTF-A and p-ERK was completely opposite (Fig. 1b and Fig. 4b). On the contrary, in HaCaT cells, activated ERK can phosphorylate MRTF-A because of absolute cytoplasmic distribution of MRTF-A and p-ERK (Fig. 3a and Fig. 4c), and ERK phosphorylation of MRTF-A plays a role in the regulation of its subcellular localization (Fig. 4e and Supplementary Figure 3). However, in HAoECs, there is not enough physical space for phosphorylation of MRTF-A by ERK because MRTF-A is constitutively localized in the nucleus (Fig. 1b,2b, and Supplementary Figure 1a, c). Furthermore, exogenously expressed pseudo-phosphorylated mutant (MRTF-A S/D) was primarily localized in both the nucleus and the cytoplasm (Fig. 4d). These findings suggest that reduced ERK phosphorylation of MRTF-A could be a partial but not complete basis for constitutive nuclear localization of MRTF-A in HAoECs. Further studies focusing on the low affinity of G-actin for MRTF-A in HAoECs is necessary to reveal a mechanism for this cellular event.

We also found that MRTF-A/B were constitutively localized in the nucleus in human umbilical vein endothelial cells (Huvecs). Like HAoECs, treatment with LatB did not significantly affect their nuclear localization (Supplementary Figure 7). These findings suggest that the constitutive nuclear localization of MRTF-A/B in Huvecs might be regulated in a similar manner in HAoECs.

Experimental evidence suggests that myocardin and MRTF-A negatively regulate NF−κB-dependent gene expression by forming complexes with NF−κB p65 in TNF−α- or LPS-stimulated cells^33^,^34^. Our novel finding is that in HAoECs, MRTF-A/B downregulation did not induce additional nuclear import of NF−κB p65, but alleviated their negative effects against constitutively nuclear localized NF−κB p65, resulting in upregulation of ICAM-1 (Fig. 6 and Supplementary Figure 6). This is strongly supported by the results shown in Fig. 5d; NF−κB p65 knockdown with siRNA prevented induction of ICAM-1 expression in MRTF-A/B-depleted HAoECs. Thus, MRTF-A/B suppressed the activation of NF−κB under nonstimulating conditions. In HAoECs, MRTF-A/B do not function as a coactivator(s) for SRF in HAoECs because expression of EMT markers was not detected. Tang *et al.* reported that NF−κB p65 inhibits myocardin function by physically interacting with myocardin^33^. Therefore, we speculate that complexes between MRTF-A or MRTF-B and NF−κB p65 may inhibit transcription mediated by SRF and MRTF-A/B. In future study, we will reveal such inhibitory mechanism.

Fang *et al.* reported that oxLDL-stimulated HAoECs overproduced MRTF-A, which resulted in ICAM-1 upregulation^26^. Their conclusions were completely opposite to our finding that overproduction of MRTF-A reduced TNF−α-induced ICAM-1 expression (Fig. 5e). Considering the results in previous reports that the interaction between NF−κB p65 and myocardin or MRTF-A plays an inhibitory role in NF−κB-mediated signaling^33^,^34^, it is intriguing that the interaction between MRTF-A and NF−κB p65 enhances transcription mediated by an NF−κB pathway. The reasons for such differences are not clear. One possibility is a difference in the source of HAoECs; we used HAoECs from thoracic aorta in this study, but Fang *et al.* used HAoECs from some different arteries including coronary artery, pulmonary artery, and microvascular cardiac cells^26^. Thoracic aorta and these cardiac arteries are not differentiated from common progenitors, thus suggesting that the difference in their progenitor cells may lead to such inconsistent findings. Further studies will be necessary to clarify how such conflicting results can be obtained.

Inflammatory cell adhesion molecules, chemokines, and cytokines are closely related to the pathogenesis of vascular damage. After vascular occlusion, the upregulation of cytokines including IL-1 and IL-6 promotes the expression of ICAM-1, P-selectin, and E-selectin in the vascular endothelium, and these inflammatory signals further promote vascular endothelium dysfunction^37^. MRTF-A/B downregulation in growth factor-depleted vascular endothelium may be, in part, a cause of these disorders. This possibility is supported by our findings that MRTF-A/B depletion results in an increase in the cell surface expression of ICAM-1 and interactions between HAoECs and leukocytes (Supplementary Figure 5).

## Materials and Methods

### Reagents and antibodies

LPS and TNF−α were from Sigma (St. Louis, MO) and Promega (Madison, WI), respectively. LatB and Y27632 were from Caymen Chemical (Ann Arbor, MI). Antibodies used in this study are as follows: anti-CD34 antibody (Nichirei Biosciences, Tokyo, Japan); anti-MRTF-B (MKL2), anti-β−actin, and anti-α−tubulin antibodies and anti-Flag M2 gel (Sigma); anti-HA affinity matrix and anti-HA (3F10) antibody (Roche Applied Science, Mannheim, Germany); anti-DYKDDDDK (anti-Flag) antibody (Trans Genic, Kobe, Japan); anti-VE-Cdh and anti-VCAM-1 antibodies (Cell Signaling Technology, Beverly, MA); anti-glyceraldehyde-3-phosphate dehydrogenase (GAPDH) antibody (Ambion, Austin, TX); anti-CRM1 (abcam, Cambridge, MA); anti-importin α1 (Life Technologies, Carlsbad, CA); anti-importin α2 (BD Transduction Laboratories, Franklin Lakes, NJ); anti-importin α4 (GeneTex, Irvine, CA): and anti-eNos, anti-histone H2B, anti-ICAM-1, anti-importin β1, anti-NF−κB p65, anti-SRF, anti-ERK, anti-p-ERK antibodies, anti-cofilin, anti-phospho-cofilin (Santa Cruz Biotechnology, Santa Cruz, CA). Phalloidine was conjugated to Alexa 568 and secondary antibodies were conjugated to Alexa 568 or Alexa 488 (Molecular Probes, Eugene, OR). The phospho-specific MRTF-A (serine 454 MAL met/serine 546 MAL fl) antibody was described previously^16^. We produced a polyclonal antibody against MRTF-A. In brief, a peptide comprising of amino acids 714-728 of mouse MRTF-A (MAL met) (GenBank: BC050941.1) was injected into a rabbit and the antiserum was subjected to affinity purification.

### Plasmids

Construction of the plasmids used in this study was previously described^12^,^14^. The expression plasmids for MRTF-A S/A and MRTF-A S/D were constructed by PCR-mediated mutagenesis. cDNA of mouse full-length NF−κB p65 (NCBI Reference Sequence: NM_009045) was amplified by RT-PCR and inserted into a mammalian expression plasmid, pCS2+, with an HA tag at the N-terminus. The sequences of newly constructed plasmids were confirmed.

### Histological examination

This study was conducted according to the guidelines laid down in the Declaration of Helsinki. All experimental protocols were approved by the Ethical Committees of Iwate Medical University and written informed consents were obtained. To examine the expression of MRTF-A/B in normal human artery, renal tissues obtained from 10 autopsy cases were used. The tissues were fixed in 10% buffered formalin solution and were embedded in paraffin. Serial 4 μm sections were used for HE staining and immunostaining. The sections were treated with antigen retrieval method using citrate buffer (pH 7.0) and were blocked by 3% hydrogen peroxide in methanol. Thereafter, the sections were incubated with primary antibody for 24 h and then with peroxidase-conjugated secondary antibody (Histofine Simple Stain MAX-PO, Nichirei Biosciences INC, Japan) for 1 h. Immunodetection was performed using Liquid DAB + Substrate Chromogen system (Dako, Glostrup, Denmark).

### Cell culture, transfection, and immunocytochemistry

Primary cultured HAoECs (C-12271) and HaCaT cells were purchased from PromoCell (Heidelberg, Germany) and CLS Cell Lines Service GmbH (Epplheom, Germany), respectively. Culture plates and coverslips used for HAoECs were coated with collagen type-I (Nitta Gelatin Inc., Osaka, Japan). HAoECs (passaged 2–6 times) were cultured in HEC-C1 complete growth medium containing 10% fetal calf serum, acidic fibroblast growth factor, and heparin^38^ (Research Institute for the Functional Peptides, Yamagata, Japan) or 0.1× HEC-C1 medium [low-serum medium; HEC-C1 medium diluted 10-fold with MCDB107 basal medium (Research Institute for the Functional Peptides)]. NIH3T3 cells and HaCaT cells were cultured in Dulbecco’s modified Eagle’s medium supplemented with 10% fetal calf serum or HEC-C1 medium. HAoECs and HaCaT cells were transfected with the indicated expression plasmid using Lipofectamine 3000 (Invitrogen, Carlsbad, CA) and ViaFect (Promega), respectively. siRNAs against human MRTF-A (Hs_MKL1_6467), MRTF-B (Hs_MKL2_6806), NF−κB p65 (Hs_RELA_1091), and a scrambled siRNA for control experiments were purchased from Sigma. siRNA transfection was performed using Lipofectamine RNAiMAX (Invitrogen).

Fluorescent images of immunocytochemistry were acquired using a Biorevo BZ-9000 fluorescence microscope (Keyence, Osaka, Japan). MRTF-A/B expression patterns were categorized into three groups: nuclear-specific localization (N); diffuse distribution in the nucleus and the cytoplasm (NC); and cytoplasmic localization (C)^14^,^15^. For each experiment (at least 3 independent experiments), 100–200 cells were analyzed. The proportions of cells that exhibited the respective expression patterns were determined (means ± SEMs).

### RT-PCR analyses

Total RNA was extracted from HAoECs cultured under the indicated conditions. MRTF-A/B and ICAM-1 mRNA expressions were quantified by quantitative RT-PCR normalized to GAPDH mRNA expression as described previously12. The specific primers to analyze the expression levels of MRTF-A isoforms are as follows: MAL fl sense primer, CCTCCACTTAGTGAGCGGAAG; MAL BSAC (MKL1 transcript variant X1) sense primer, ATGACTCTACTGGAACCTGAG; MKL1 transcript variant X2 sense primer, ATGAGAAGAGAGGGCAGCACTG; MRTF-A common antisense primer, GCAGTGGTTCGCTGACTCGG. The specific primer sets used are as follows: MRTF-A common sense primer, CCGAGTCAGCGAACCACTGC; MRTF-A common antisense primer, CCGAGTCAGCGAACCACTGC; MRTF-B sense primer, AAAGTTAGTGAATCGCCATCTCC; MRTF-B antisense primer, AGTGGCTTGAATGGTGCAGGC; ICAM-1 sense primer, GCTGGTGACATGCAGCACC; ICAM-1 antisense primer, CTCCTCACCAGCACCGTGG; GAPDH sense primer, CTGGTAAAGTGGATATTGTTG; GAPDH antisense primer, CATGAGTCCTTCCACGATAC. The expression levels of these mRNAs were also analyzed by quantitative real time RT-PCR using SYBR GreenER qPCR SuperMix (Invitrogen). These primers used are as follows: MRTF-A sense primer, CCTCACCGTGACCAATAAGAATGC; MRTF-A antisense primer, GCTGGGACGAGGGCTGCT; MRTF-B sense primer, CAGTCAGCCTCAACAAGTCAGAA; MRTF-B antisense primer, GGCAGGATGTCTCTGATATGGAAG; ICAM-1 sense primer, GCAACCTCAGCCTCGCTATG; ICAM-1 antisense primer, GGACACAGATGTCTGGGCATTG; GAPDH sense primer, ACTCCTCCACCTTTGACGCTG; GAPDH antisense primer, GCCAAATTCGTTGTCATACCAGGAA.

### ICAM-1 cell surface expression and leukocyte adhesion assay

ICAM-1 cell surface expression on HAoECs was analyzed by flow cytometry. HAoECs were transfected with the indicated siRNA and were cultured for 2 days. These cells were then incubated on ice with 1 μg/ml of a mouse anti-ICAM-1 monoclonal antibody (clone HA58, BioLegend, San Diego, CA) or a control mouse antibody, followed by an FITC-conjugated anti-mouse antibody. Cells were analyzed with a FACS Calibur (BD Bioscience, San Jose, CA). Obtained data were analyzed using CELL Quest software (BD Bioscience). For cell adhesion assay, HAoECs transfected with the indicated siRNA were similarly cultured in a 96-well plate. Cells were then incubated in the presence of 10  μg/ml of either a control antibody or the anti-ICAM-1 antibody for 30 min. Jurkat T cells were labeled with 5 μM 5-chloromethylfluorescein diacetate (Life Technology, Carlsbad, CA) for 30 min at 37 °C, and then were incubated with 100  ng/ml of phorbol 12-myristate 13-acetate for 30 min. They were resupsended in HEC-C1 medium, and were added to the HAoEC cultures (1 × 10^5^ cells/well). Cells were allowed to adhere for 30 min at 37 °C. After removing nonadherent cells, cells were lysed with 1% Nonidet P-40 (NP-40)/PBS. The fluorescence intensity of each well was measured at 492 nm excitation and 517 nm emission using a microplate reader (SH-9000, Corona Electric, Ibaraki, Japan).

### ChIP assay

ChIP assays were performed using ChIP assay kits (Merck Millipore, Billerica, MA) according to the manufacturer’s instructions. DNAs isolated from input chromatin fragments and precipitated chromatin fragments by a control antibody or anti-NF−κB p65 antibody were subjected to PCR and quantitative real time PCR using primers that flanked the NF−κB-binding site of the human ICAM-1 gene promoter. These primer sequences used are as follows: sense primer, CCTGGAGTCTCAGTTTACCGC; antisense primer, AACTCTGAGTAGCAGAGGAGC.

### Protein–protein interaction analyses

MRTF-A- CRM1 interactions were performed as previously described^14^,^15^. The interactions between MRTF-A/B and NF−κB p65 were examined as follows. Flag-tagged MRTF-A/B and HA-tagged NF−κB p65 proteins were prepared using a TNT SP6 high-yield expression system based on an optimized wheat germ extract (Promega). The IP buffer mixtures (500 μl total volume) containing Flag-MRTF-A or Flag-MRTF-B proteins (15 μl) and HA-NF−κB p65 proteins (35 μl) were subjected to IP analyses as described previously^12^. The IP buffer included 20 mM Tris–HCl, pH 7.5, 0.5% NP-40, 150 mM NaCl, 1 mM EDTA, 50 mM NaF, 10 mM β−glycerophosphate, and proteinase inhibitors (complete Mini; Roche Applied Science). Proteins in immunoprecipitates were detected by IB with the indicated antibodies. For these analyses, 3.3% of the input proteins and 22.2% of the IP proteins were subjected to IB, respectively. The IP/IB analyses using whole cell extracts were similarly performed. Quantification of the respective IP/IB signal intensities was performed using NIH ImageJ software.

### Preparation of cell extracts

Whole cell extracts and subcellular proteomes were prepared as described previously^39^,^40^. The cytoplasmic and nuclear fractions were subjected to IB with the indicated antibodies. The respective IB signal intensities were quantified using NIH ImageJ software.

### DNA affinity binding assay

A biotinylated probe for the human ICAM-1 gene promoter region containing the NF−κB-binding site was coupled with Streptavidin M-280 Dynabeads (Invitrogen, Carlsbad, CA) according to the manufacturer’s instructions. The Dynabeads-NF−κB oligos obtained were mixed with the following *in vitro* translated proteins and were incubated with rotation for 2 h at 4 °C: HA-NF−κB p65 proteins (35 μl) alone or with Flag-MRTF-A or Flag-MRTF-B proteins (15 μl) in gel-shift binding buffer^3^ containing 5 mM MgCl_2_, 5 μg of poly(dI-dC), 20 μg of herring sperm DNA, and 0.5% NP-40 (200 μl total volume). After washing, probe-bound proteins were analyzed by IB with the indicated antibodies. DNA affinity binding assay was performed similarly using the indicated HAoEC whole cell extracts. Quantification of the respective pull-down/IB signal intensities was performed using NIH ImageJ software.

### Actin fractionation

The F- and G-actin pools were separated by centrifugation^41^. The supernatant (G-actin) and pellet (F-actin) fractions were subjected to IB with anti-β−actin antibody.

### Statistical analysis

Results are given as means and standard errors. Statistical comparisons were made by two-tailed paired student t-tests. The significance level is set at 0.05.

## Additional Information

**How to cite this article**: Hayashi, K. *et al.* A novel inhibitory mechanism of MRTF-A/B on the ICAM-1 gene expression in vascular endothelial cells. *Sci. Rep.* **5**, 10627; doi: 10.1038/srep10627 (2015).

## Supplementary Material

Supporting Information
