# Supplementary material for: A novel inhibitory mechanism of MRTF-A/B on the ICAM-1 gene expression in vascular endothelial cells
Source: Sci Rep. 2015 May 29;5:10627. doi: 10.1038/srep10627 (PMC4448521; doi:10.1038/srep10627)
Supplement: Supporting Information [file srep10627-s1.pdf]

Supplementary information

A novel inhibitory mechanism of MRTF-A/B on the ICAM-1 gene expression in vascular endothelial cells

Ken'ichiro Hayashi, Toshiyuki Murai, Hiroki Oikawa, Tomoyuki Masuda, Kazuhiro Kimura, Susanne Muehlich, Ron Prywes, and Tsuyoshi Morita

Supplementary Figure Legends

Supplementary Figure 1. Effects of Dulbecco's modified Eagle's medium or **latrunculin B on the nuclear accumulation of MRTF-A/B in HAoECs.** (a and b) HAoECs were cultured in HEC-C1 medium. For the last 24 h, they were cultured in Dulbecco's modified Eagle's medium supplemented with 10% or 0.3% fetal calf serum. Cells were stained either with anti-MRTF-A antibody or anti-MRTF-B antibody (red) and Hoechst 33258 (blue). (c and d) HAoECs were cultured in HEC-C1 medium. For the last 15 min, they were treated with vehicle or 0.5 μM latrunculin B (LatB). Cells were stained either with anti-MRTF-A antibody or anti-MRTF-B antibody (green), phalloidine-Alexa 568 (red), and Hoechst 33258 (blue). Representative images from at least three independent experiments are shown. Images were quantified as described in the legend for Figure 1. Statistical differences were calculated using student's t-test.

Supplementary Figure 2. Effects of MRTF-A phosphorylation by ERK on the interaction between MRTF-A and CRM1. (a) Differences in the binding levels of mutant and wild-type (wt) MRTF-As for CRM1. MRTF-A S/A and MRTF-A S/D indicate MRTF-A mutants at the ERK phosphorylation site [un-phosphorylated mutant (S/A) and pseudo-phosphorylated mutant (S/D)]. GST-Flag-RanQ69L is the glutathione S-transferase (GST)-fusion protein of the Flag-tagged constitutively active form of human Ran (constitutively GTP-bound Ran). Complex formation between HA-CRM1 and each of mutant and wt Flag-MRTF-As was examined by IP/IB analyses using the indicated *in vitro* translated proteins and purified GTP-bound GST- Flag -RanQ69L. Mixtures of the indicated proteins were immunoprecipitated with a control (cntl) gel or anti-HA affinity (HA) gel as described in Materials and Methods. The

resulting immunoprecipitates were subjected to IB with the indicated antibodies. (b and c) Effect of unpolymerized mutant β−actin (Myc-tagged β−actin R62D)[1] on the interaction between CRM1 and MRTF-A wt (b) or MRTF-A S/D (c). The indicated proteins were mixed with 10 μl (+) and 20 μl (++) of *in vitro* translated Myc-tagged β−actin R62D protein, and the interactions were analyzed by IP/IB analyses as described earlier. Positions of molecular weight markers (kDa) are indicated between the IB panels. IB signal intensities for CRM1-bound MRTF-As were quantified. Percentages indicate the relative binding levels of the respective MRTF-As to CRM1 normalized by the level of MRTF-A wt (a and b) or MRTF-A S/D in the absence of β−actin R62D (c), which was set at 100% (means ± SEMs of three independent experiments). Statistical differences were calculated using student's t-test. *P = 0.0001 and **P = 0.0057 versus the value of MRTF-A wt (a). *P = 0.0020 and **P = 4.2510×10^{-5} *versus* the value of MRTF-A wt in the absence of β−actin R62D (b).

Supplementary Figure 3. Effects of U0126 on the subcellular localization of MRTF-A in HaCaT cells. HaCaT cells were cultured in HEC-C1 medium. For the last 24 h, they were treated with vehicle or 10 μM U0126. Cells were stained with anti-MRTF-A antibody (red) and Hoechst 33258 (blue). Representative images from at least three independent experiments are shown. Images were quantified as described in the legend for Figure 1. Statistical differences were calculated using student's t-test.

Supplementary Figure 4. Mutual dependence of MRTF-A/B for their expressional regulation. HAoECs were transfected with the indicated siRNA and were cultured for 2 days in HEC-C1 medium. For the last 24 h, they were cultured in in 0.1× HEC-C1 medium. Whole cell lysates for the respective HAoECs were subjected to IB with the indicated antibodies (left column). Culture conditions are shown at the bottom of the IB panel. α−tubulin was used as a loading control. Quantification of the IB signal intensities of MRTF-A and MRTF-B was performed with the NIH ImageJ software (right column). The levels of MRTF-A/B proteins in control (cntl) siRNA-transfected HAoECs cultured in 0.1× HEC-C1 medium were set at 100% (means ± SEMs of three independent experiments). Statistical differences were calculated using student's t-test. *P = 1.2273×10^{-6}, **P = 3.2500×10^{-5}, and $^{\#}$P =

8.5851×10^{-8} *versus* the value from control siRNA-transfected cells (b, upper panel). $*P = 1.5988 \times 10^{-7}$, $**P = 2.6179 \times 10^{-8}$, and $^{\#}P = 6.2818 \times 10^{-12}$ *versus* the value from control siRNA-transfected cells (b, lower panel).

Supplementary Figure 5. ICAM-1 cell surface expression and leukocyte adhesion assay. (a and b) HAoECs were transfected with control (cntl) siRNA (thin lines) or either anti-MRTF-A siRNA or anti-MRTF-B siRNA (thick lines). At 2 days after transfection, ICAM-1 protein expression levels on cell surfaces were analyzed by flow cytometry as described in Materials and Methods. Gray-filled profiles indicate isotype-matched controls. (c) HAoECs were transfected with the indicated siRNA. At 2 days after transfection, the interactions between HAoECs and Jurkat T cells were assessed as described in Materials and Methods. Percentages indicate the proportions of adherent cells (means ± SEMs of three independent experiments). Statistical differences were calculated using student's t-test. $*P = 3.0154 \times 10^{-6}$ and $**P = 0.0014$ *versus* the value from control siRNA-transfected cells with a control antibody.

Supplementary Figure 6. Inhibition of NF−κB p65 binding to the human ICAM-1 gene promoter region by MRTF-A/B. (a) ChiP assays were performed with a control antibody (cntl-Ab) or anti-NF−κB p65 antibody as described in Materials and Methods. PCR products were sampled after 32 and 34 cycles and were separated on 1.2% agarose gels. The upper and lower panels show the PCR products after 34 cycles and the results of ChiP-quantitative real-time RCR (means ± SEMs of three independent experiments), respectively. (b) IP analyses for detection of the interaction between endogenous NF−κB p65 and MRTF-A or MRTF-B in HAoECs. Whole cell extracts (WE) from cultured HAoECs were subjected to IP/IB analysis with a control antibody or anti-NF−κB p65 antibody as described in Materials and Methods. (c and d) DNA affinity binding assay using *in vitro* translated proteins (c) or HAoEC WE (d). Schematic diagram of Dynabeads-NF−κB oligos for pull-down assay is shown (c, upper panel). Bold letters with underlines indicate the NF−κB-binding site. Streptavidin M-280 Dynabeads (black circles) were attached to the 5'-ends of this probe. The Dynabeads itself was used in a control experiment. Mixtures of the indicated *in vitro* translated proteins or HAoEC WE in the absence or presence of free NF−κB oligos (400 pmol) were subjected to IP (c, lower right panel) or pull-down assay (c, lower left

panel) as described in Materials and Methods. In the case of the pull-down assay using HAoEC WE, α–tubulin was used as an input control (d). Probe-bound proteins were analyzed by IB with the indicated antibodies. Quantification of the pull-down/IB signal intensities was quantified as described in Materials and Methods. Percentages indicate the relative binding levels of NF–κB p65 for the probe normalized by the levels in the absence of MRTF-A/B (c) or in HAoECs transfected with cntl siRNA (d), which was set at 100% (means ± SEMs of three independent experiments). Statistical differences were calculated using student's t-test. *P = 0.0012 and **P = 0.0014 (a) and $^{\#}P = 7.9141 \times 10^{-6}$ and $^{\#\#}P = 1.8249 \times 10^{-7}$ (d) *versus* the values from control siRNA-transfected cells/IP with NF–κB p65 antibody or pull-down with Dynabeads-NF –κB oligos.

Supplementary Figure 7. Effects of latrunculin B on the nuclear accumulation of MRTF-A/B in Huvecs. Huvecs (Promo Cell C-12203) were cultured in HEC-C1 medium. For the last 15 min, they were treated with vehicle (LatB-) or 0.5 μM LatB (LatB+). Cells were stained either with anti-MRTF-A antibody or anti-MRTF-B antibody (green), phalloidine-Alexa 568 (red), and Hoechst 33258 (blue). Representative images from at least three independent experiments are shown. Images were quantified as described in the legend for Figure 1.

Reference

1. Posern, G., Sotiropoulos, A. & Treisman, R. Mutant actins demonstrate a role for unpolymerized actin in control of transcription by serum response factor. *Mol Biol Cell* **13**, 4167–4178 (2002).

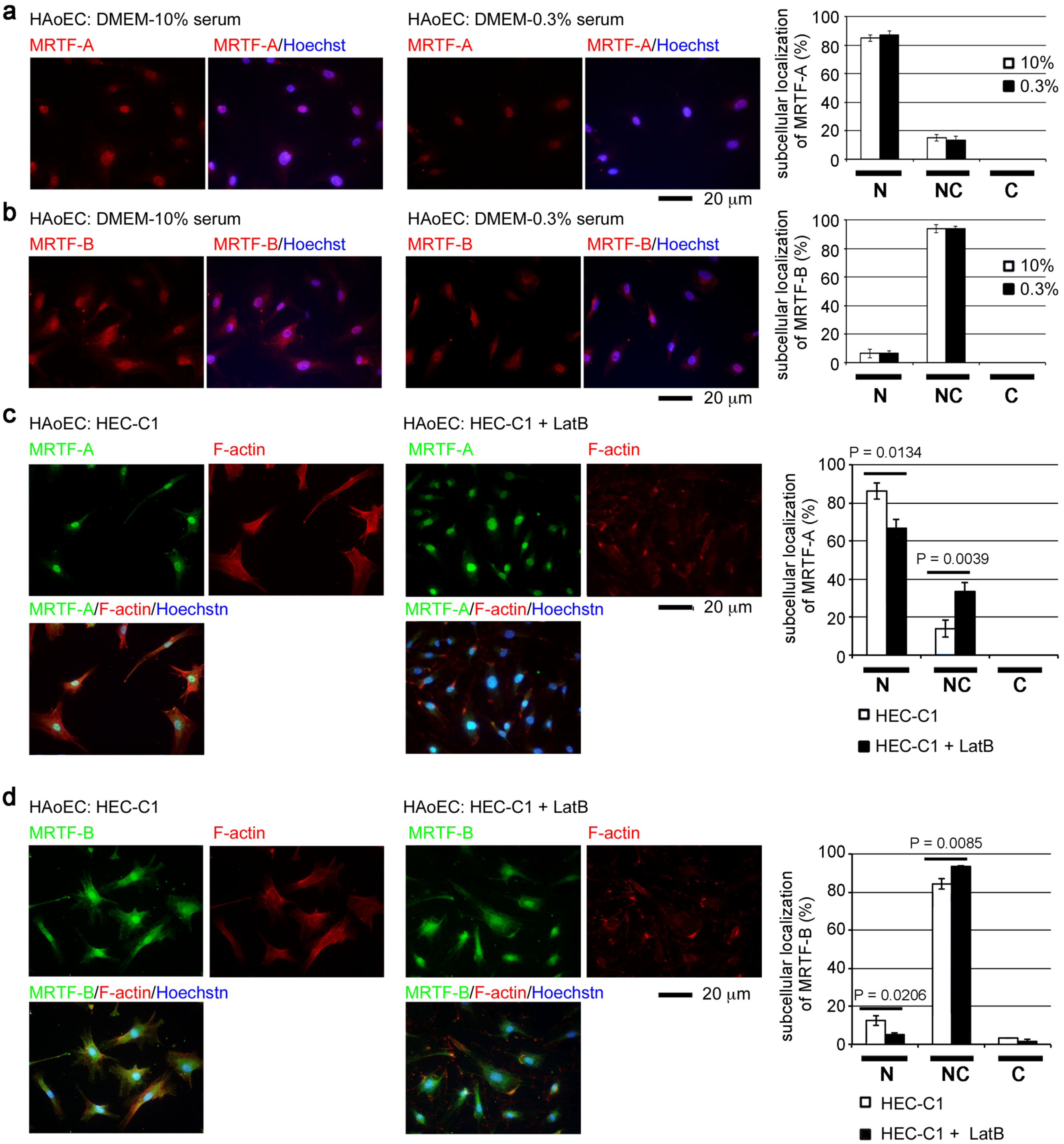

Suppl Figure 1

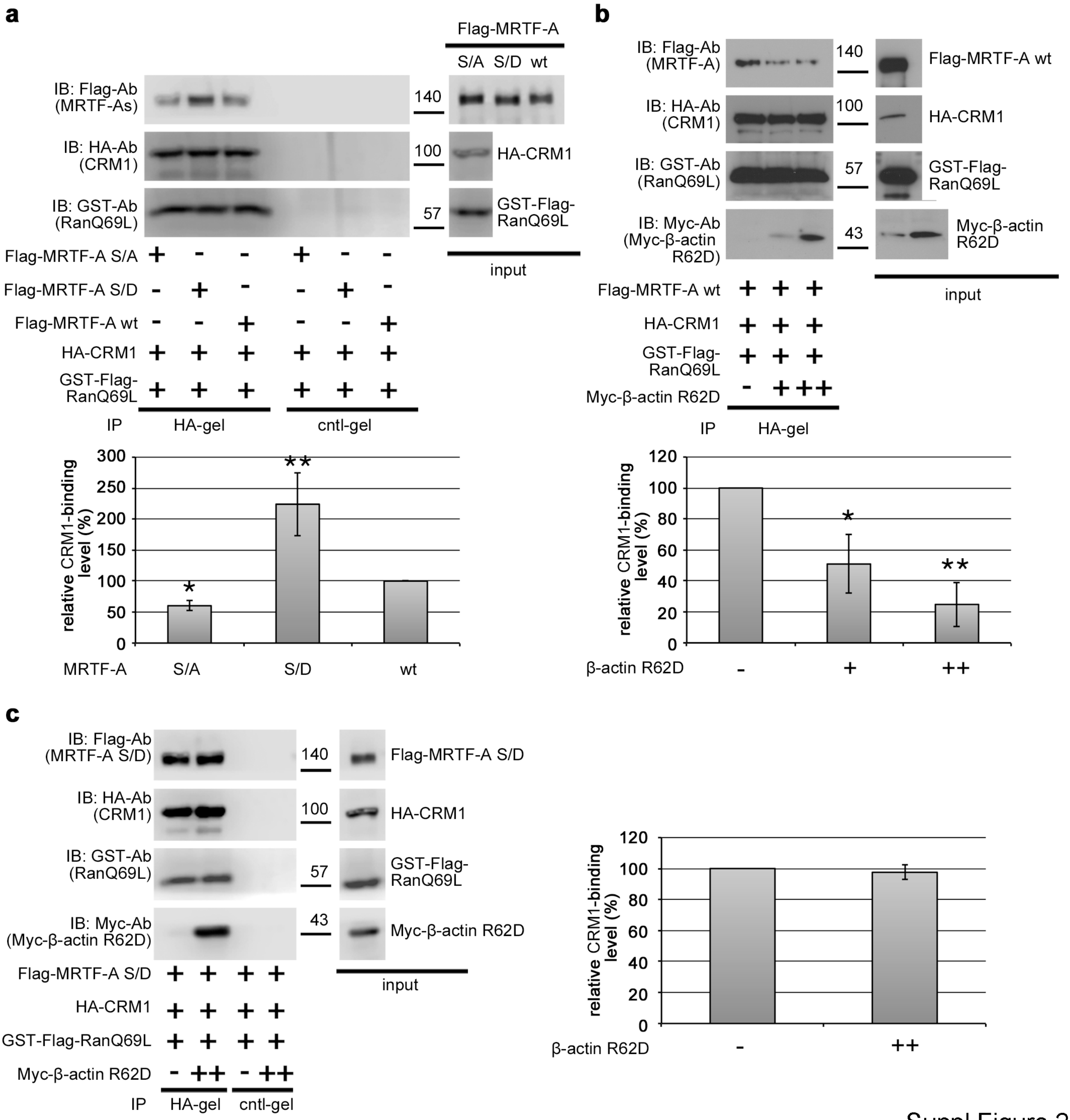

Suppl Figure 2

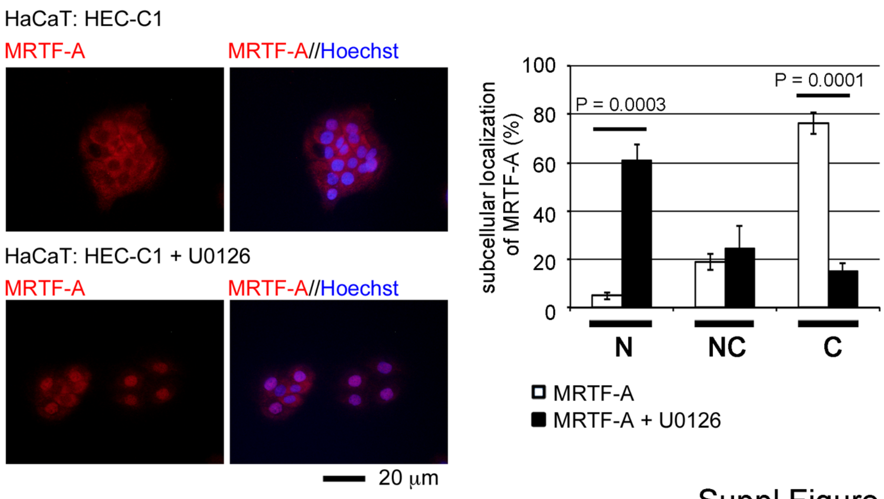

Suppl Figure 3

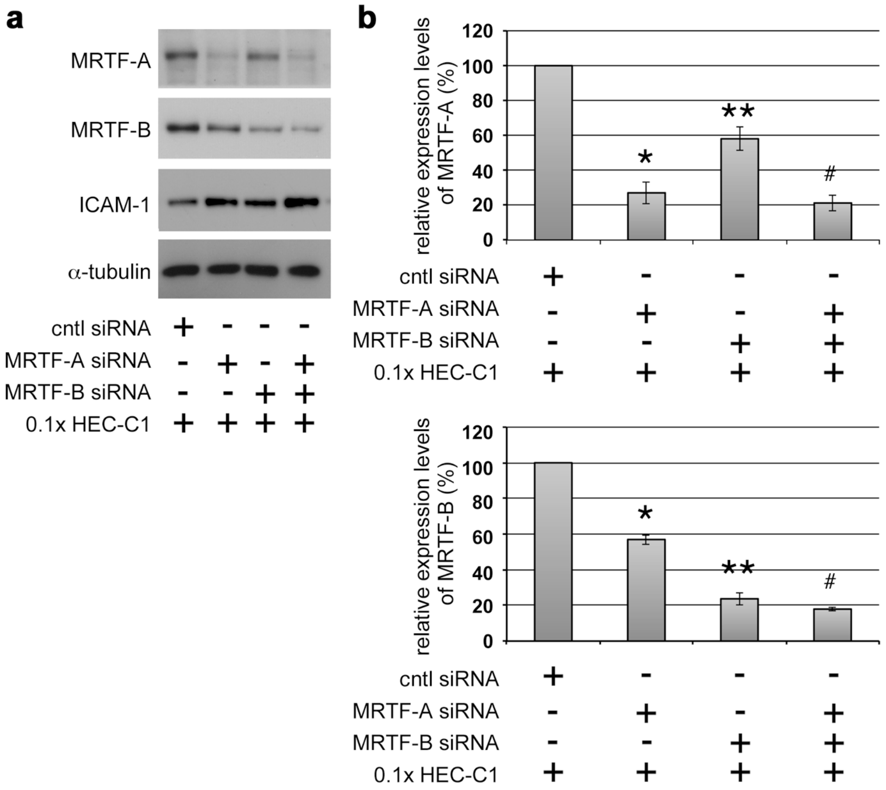

Suppl Figure 4

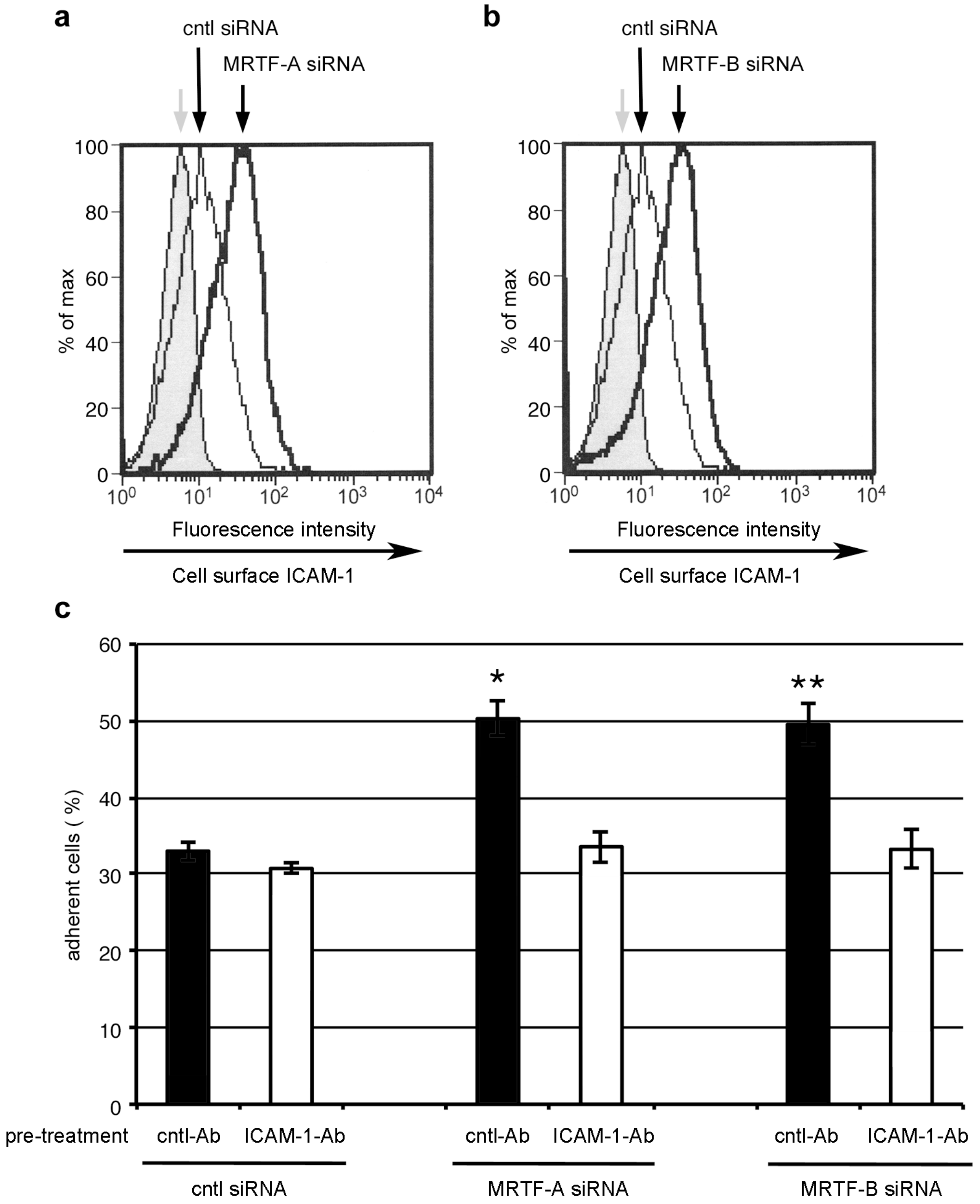

Suppl Figure 5

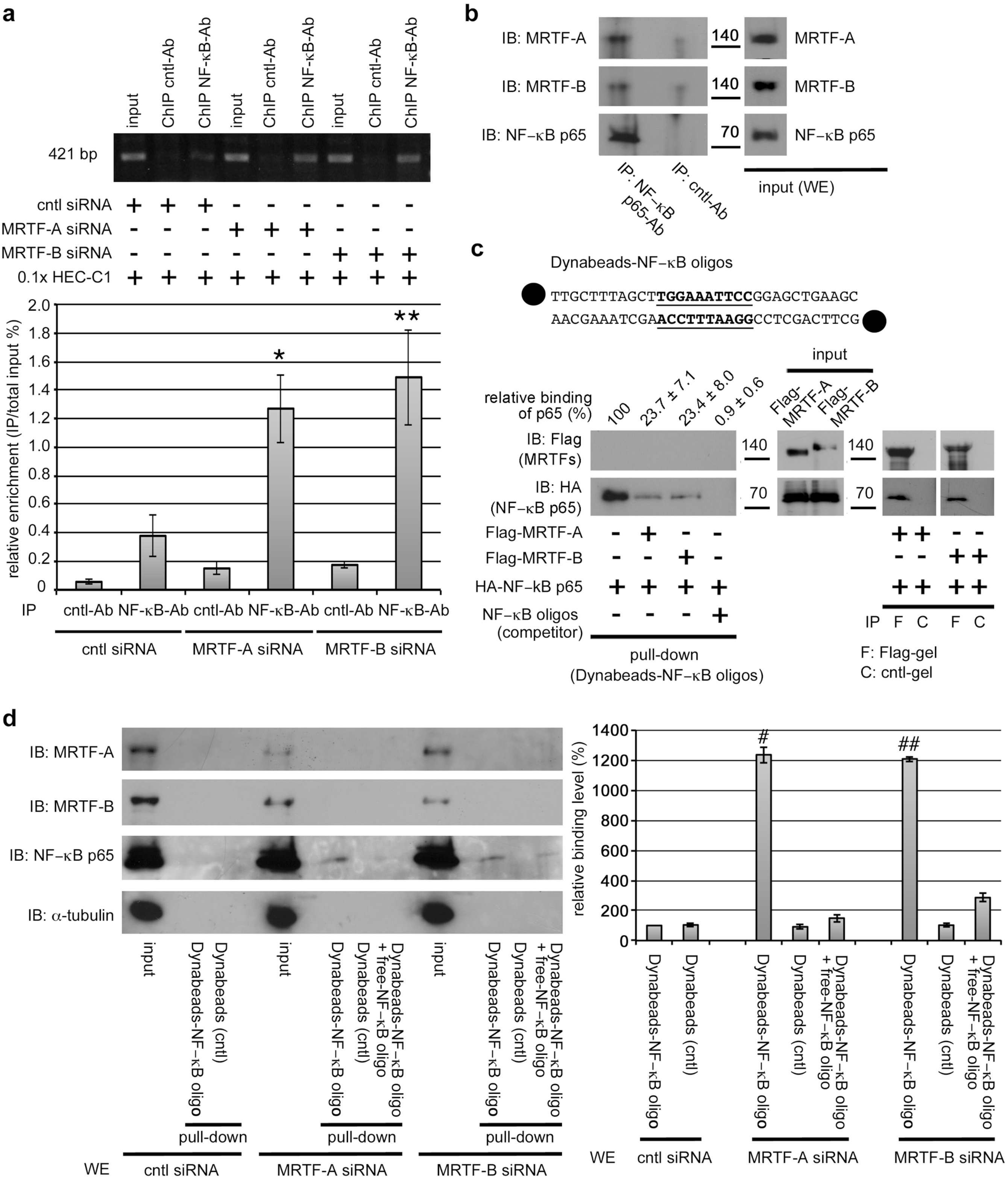

Suppl Figure 6

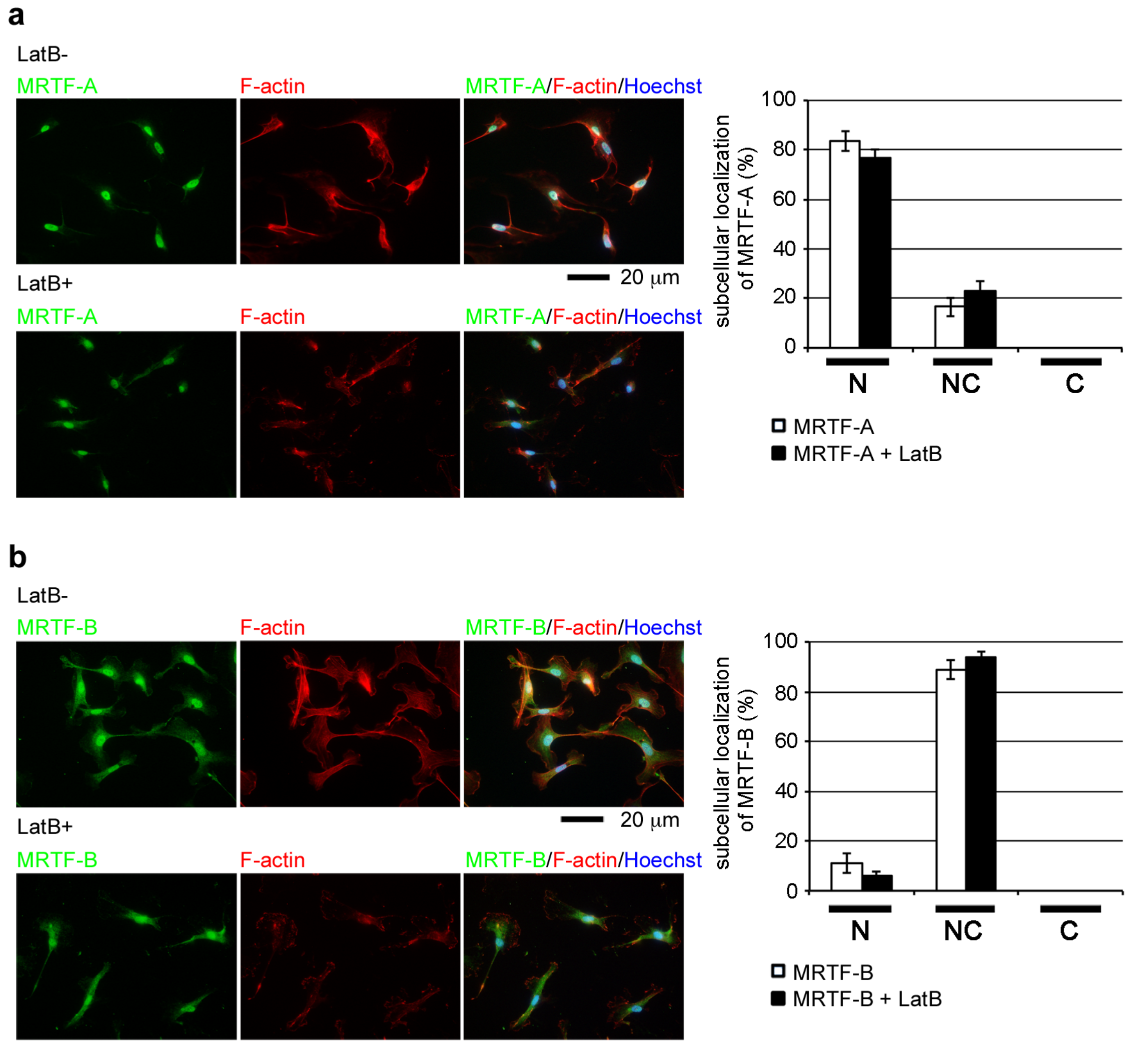

Suppl Figure 7
